# Transperitoneal laparoscopic adrenalectomy for the resection of large size pheochromocytoma: Case report and literature review

**DOI:** 10.1016/j.ijscr.2020.05.027

**Published:** 2020-05-23

**Authors:** Roosevelt Fajardo, Nicole García, Francisco Díaz

**Affiliations:** Fundación Santa Fe de Bogotá Hospital, General Surgery Department, Cra 7 # 117- 15, Bogota, Colombia

**Keywords:** ICU, Intensive Care Unit, MEN-2A/2B, Multiple Type 2 endocrine neoplasia, MRI, Magnetic Resonance Imaging, PASS Scale Score, Pheochromocytoma of the Adrenal Gland Scaled, CC, Colombian Constitution, U/S, Ultrasound, Pheochromocytoma, Neutral crest, Phenotype, Noradrenergic, Case report

## Abstract

**Introduction:**

Pheochromocytomas are rare neuroendocrine tumors that form in chromaffin cells of the neural crest during the embryological period. In the overall population, incidence rate is 0.1%; lack of early diagnosis or adequate treatment can lead to life-threatening complications. The secretion of catecholamines, such as adrenaline or norepinephrine, produces paroxysmal headache, palpitations and arterial hypertension. Secondary, hyperglycemic crises may also appear, thus contributing to misdiagnosis of diabetes mellitus or diabetic ketoacidosis. Surgery prevails as primary treatment; despite its subsequent high mortality rate of up to 50% during the intra and postoperative periods.

**Case report:**

We describe the case of a 55-year-old man, diagnosed in 2012 with arterial hypertension and almost uncontrollable labile arterial hypertension, who had been medicated with second and third-line antihypertensive drugs, but, who, nonetheless showed no clinical improvement. Biochemical profile studies showed elevated normetanephrine and metanephrine levels; and an abdominal MRI, a markedly vascularized, 72 × 62 mm diameter solid mass in the right adrenal fossa; thereby leading to its classification as a large, noradrenergic phenotype pheochromocytoma.

**Discussion:**

In 2018, at the *Fundación de Santa Fe de Bogotá Hospital*, patient underwent Transperitoneal Laparoscopic Adrenalectomy, which proceeded without complications. In the 6-month postoperative follow-up, patient remained asymptomatic for cardiovascular risk and maintained stable blood pressure within goals; consequently, antihypertensive therapy was suspended.

**Conclusion:**

Despite advances in surgical technique and perioperative management, minimally invasive surgery for resection of large pheochromocytoma is a challenging procedure, due to risk of intraoperative hemodynamic instability.

## Highlights

• Pheochromocytomas are rare tumors, in the absence of diagnosis and treatment they can lead to life-threatening complications.
• Most reported cases in the literature for laparoscopic surgery in pheochromocytomas measure under 6 cm.
• Minimally invasive surgery for resection of the pheochromocytoma larger than 6 cm remains a challenging procedure, due to the risk of intraoperative hemodynamic instability.
• In Colombia it is extremely rare to perform laparoscopic transperitoneal adrenalectomy in large size pheochromocytomas.

## Graphical abstract

**Figure:**
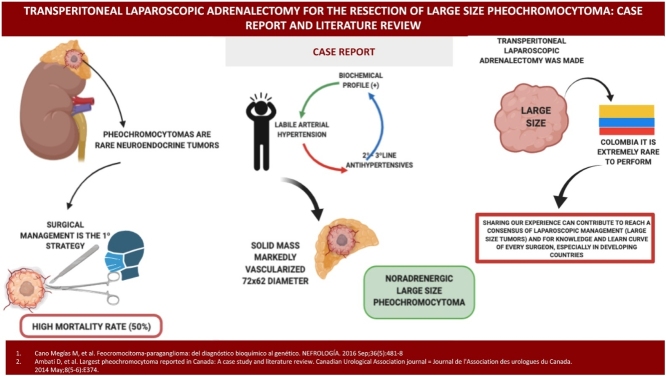

## 1 Introduction

Pheochromocytomas are neuroendocrine tumors derived from chromaffin cells in the neural crest during the embryological period. Eighty-five percent of tumors in chromaffin cells correspond to this type of tumor, the rest are paragangliomas [1,2].

An incidence rate of 0.1%, peaking in the fourth and fifth decades of life, affects the general population [3,4]. Although pheochromocytomas are rare, without adequate diagnosis and treatment, they can bring about life-threatening complications.

These tumors have the potential to secrete catecholamines, ie: adrenaline, norepinephrine and dopamine, that, in turn, produce paroxysmal headache, palpitations, arterial hypertension, diaphoresis and tachycardia. Catecholamine synthesis and secretion are among the most important factors to look for when diagnosing this type of tumor. However, the release of catecholamines may be minimal; whereas, the release of metabolites (metanephrine and normetanephrine) can be continuous [2,5,6].

Due to their unspecific clinical symptomatology, pheochromocytomas are difficult to diagnose. Furthermore, asymptomatic disease often shields these tumors, and only incidental findings in diagnostic images arouse suspicion of their presence. Given their low incidence, many physicians have never encountered this pathology [2,8].

Accurate diagnosis requires not only adequate biochemical studies, but precise tumor location, as well. Therefore, diagnostic images, which can be anatomical, to determine location and subsequent surgical approach, or functional, to evaluate the possibility of metastasis, play important roles in diagnosing and treating pheochromocytomas [1,2,9].

Complete resection is currently the only cure. However, during surgery, catecholamines are almost always released, thereby triggering hypertension or arrhythmias that may result in cardiovascular failure. The literature reports primarily on tumors less than 10 cm in diameter, and recognized laparoscopic surgery to be the most successful treatment when tumor size is less than 6 cm [22,27].

Similarly, when tumor mass is in close contact with the renal vein or inferior vena cava, surgeons should perform laparoscopic tumor resection. Despite advances in surgical procedures and perioperative management, minimally invasive pheochromocytoma resection surgery continues to be challenging; mainly due to risk of intraoperative hemodynamic instability, especially in cases where tumor diameter is greater than 6 cms [26].

This research report complies with SCARE Criteria [30].

## 2 Methods - case report

### 2.1 Background/patient description

A 55 year-old Colombian, male patient sought specialized medical care at the *Fundación Santa Fe de Bogotá Hospital.*

### 2.2 Clinical findings

We report on the case of a 55 year-old male, whose 2012 diagnosis of difficult-to-control labile arterial hypertension, which, despite treatment with second- and third-line antihypertensive medication showed no clinical improvement.

Patient’s medical background included excess body weight (BMI 22.99), sedentarism, and no specific family or personal surgical history. His pharmacological history for blood pressure included 160 mg/5 mg Valsartan/Amlodipine daily and Prazosin 1 mg every 8 h. Previous antihypertensive medications included 50 mg Losartan, every 12 h; 25 mg Hydrochlorothiazide, daily.

Physical exam blood pressure was 142/90 mmHg; abdomen showed abundant adipose panicle; palpitation revealed no masses or visceromegaly, all other physical-exam checklist specifics fell within normal limits.

### 2.3 Timeline (Table 1)

**Table 1:** Timeline.

## 3 Results

### 3.1 Diagnostic focus and assessment

Considering the difficultly of pinpointing causes for inadequate blood pressure, early diagnosis rested on secondary arterial hypertension. Subsequent epidemiological data revealed a renal ailment to be the primary cause of arterial hypertension; therefore, in 2016, patient underwent renal ultrasound and Doppler ultrasound of the renal arteries in 2016 the results of which were within normal limits. Patient´s complete 2018 abdominal ultrasound revealed a possibly neoplastic solid lesion, located in the hepatorenal cavity; however, it was not possible to determine if it was placed on the adrenal gland or on the upper pole level of the right kidney.

Accordingly, the General Surgery staff of the *Fundación Santa Fe de Bogotá* evaluated the patient. In a detailed biochemical profile, evidence appeared of high normetanephrine and metanephrine levels, thus creating suspicion of the presence of a noradrenergic phenotype pheochromocytoma (Table 2).

**Table 2:** Biochemical profile report.

BIOCHEMICAL TEST	LEVELS
Vanillylmandelic acid	15.7 mg/24 h
Metanephrines	102 mcg/24 h
Normetanephrine	6751 mcg/24 h
Total Metanephrines	6853 mcg/24 h
Dopamine	40 pg/mL
Epinephrine	5 mcg/24 h
Norepinephrine	1929 mcg/24 h
Total Catecholamines	1934 mcg/24 h
Urinary Dopamine	208 mcg/24 h

**Fig. 1:**
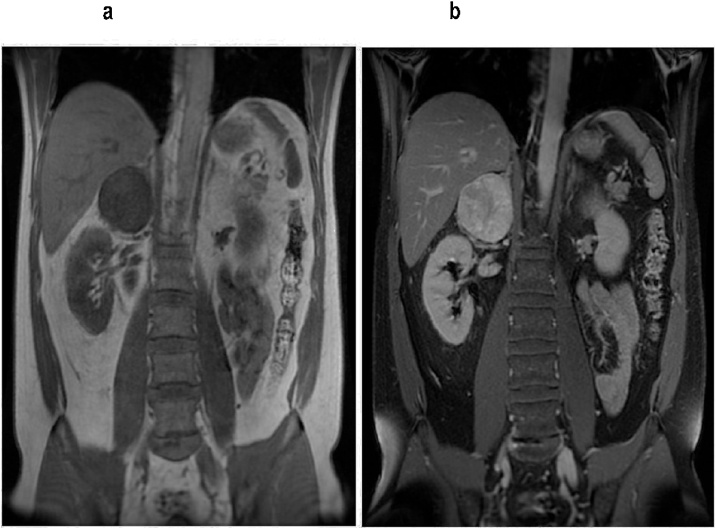
a–b: Coronal plan in abdominal Magnetic Resonance, with evidence of solid hypervascular lesion in right suprarenal cavity.

**Fig. 2:**
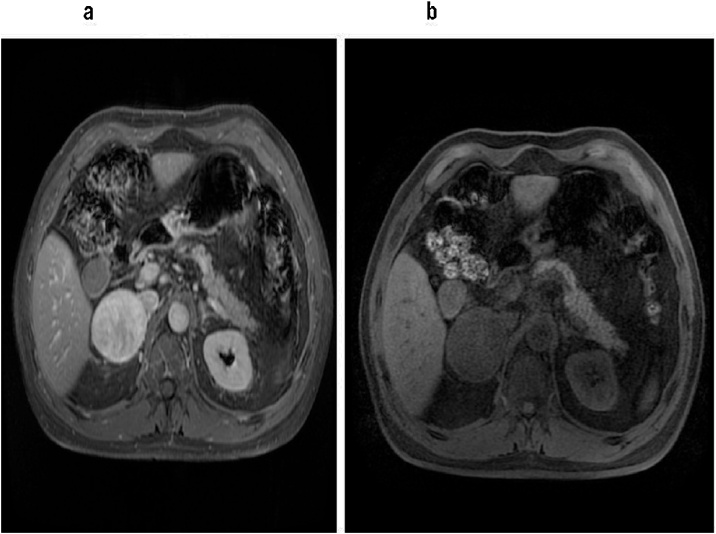
a–b: Axial plane of abdominal Magnetic Resonance with solid lesion with well-defined contour of 72 mm in right adrenal gland.

An abdominal MRI pinpointed a markedly vascularized, 72 × 62 mm, solid lesion in the right adrenal cavity, which, under contrast agent application during arterial phase, became intensely enhanced. Due to this description, examiners potentially classified the mass as a large size pheochromocytoma (Figs. 1a–b and 2a–b).

Due to the large mass size, physicians opted for transperitoneal laparoscopic adrenalectomy, which permits intraoperative conversion to open surgery if necessary, thus providing better and more surgical space. Patient agreed to scheduled surgery in September 2018 at the *Fundación Santa Fe de Bogotá Hospital*.

Before surgery commenced, anesthesiology and cardiology specialists recommended, careful use of sodium nitroprusside, in case of hypertensive crisis; possible use of esmolol for eventual supraventricular tachycardia or tachyarrhythmia; and, phenylephrine, in case of hypotension secondary to tumor resection; additionally, due to risk of hemodynamic instability following surgery, patient transfer to Intensive Care Unit.

#### 3.1.1 Surgical intervention

**Fig. 3:**
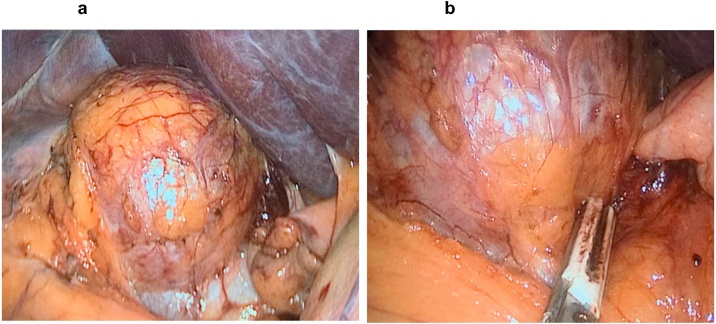
a–b: Image of intraoperative tumor, after hepatic movement.

Transperitoneal laparoscopic adrenalectomy performed by lead surgeon Roosevelt Fajardo and Francisco Díaz, second surgeon; Nicole García, XII year (2018) intern, surgical assistant.  *3.1.1.1 Medical devices*. LigaSure™ 5-mm Blunt Tip.  *3.1.1.2 Laparoscopic instrument*  *3.1.1.3 Prophylactic antibiotic*. Cephazolin  *3.1.1.4 Pharmacological therapies*. Alpha and beta-adrenergic blockage during anesthesia, deployed prior to surgical treatment.  *3.1.1.5 Surgical technique*. Under general anesthesia, patient positioned in left decubitus; # 2–5 mm and #2–12 mm trocars put in place; liver lifted in right hypochondrium; complete dissection until vena cava and right renal vein identified; following right adrenal vein identification, hemolock fixation; using Ligasure, tumor mass dissected; revealing direct relationship with vena cava and renal vein; through an access port, tumor mass extracted and placed in a sample collection bag; cavity closed in planes (Fig. 3a–b).

**Fig. 4:**
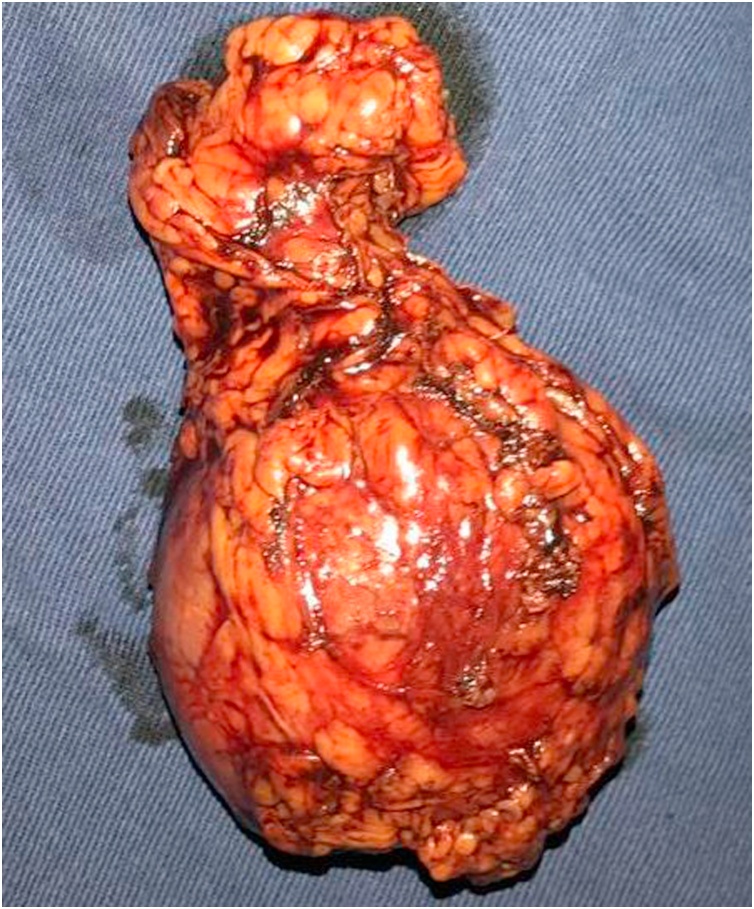
Pheochromocytoma extracted in surgery room.

Surgery proceeded without complications; no significant blood loss or surgical wound complications occurred; patient transferred to ICU following surgery Pheochromocytoma diagnosis confirmed on the basis of surgical photographs and anatomopathological report on 5.3 × 5.5 cm rounded tumor lesion, PASS Scale Score of 5/20, related to high aggressive-behavior risk (Fig. 4).

### 3.2 Follow-up and outcomes

At one month postop (October 2018), surgical specialist examined patient at the *Fundación Santa Fe de Bogotá Hospital* to ensure surgical wound was healing properly. Cardiology and internal medicine specialists also examined patient and verified favorable progress in adequate blood pressure and heart rate within, asymptomatic cardiovascular condition, and no need for anti-hypertension management. At subsequent six.-month follow-up, blood pressure remained stable, requiring no pharmacological treatment.

## 4 Discussion

Pheochromocytomas are neuroendocrine tumors that form in chromaffin cells of the neural crest during the embryological period. This type of tumor accounts for 85% of all tumors in chromaffin cells, the remainder corresponds to paragangliomas [1,2].

In the general population, these rare tumors have an estimated incidence rate of 0.1%, which peaks during fourth and fifth decades of life; and their presence, can lead to life-threatening complications if diagnosis and treatment do not occur in a timely manner. [3,4].

In 1886, Frankel considered pheochromocytoma to be either a sporadic pathology or part of associated hereditary syndromes, including Hippel - Lindau Syndrome, Type 1 Neurofibromatosis or Multiple Endocrine Neoplasia Type 2 (MEN-2A/2B) [5,7].

Pheochromocytoma can potentially secrete catecholamines, such as adrenaline, norepinephrine and dopamine, which, in turn, induce paroxysmal headache, palpitations and arterial hypertension; and even diaphoresis and tachycardia. Nevertheless, only 30%–40% of patients will clinically manifest any of these symptoms; furthermore, between 5 and 55% of patients will continue to have normal blood pressure [2,5,6].

The literature describes patients whose arterial hypertension was associated with pregnancy and dilated cardiomyopathy, secondary to production of catecholamines. In other catecholamine-related cases, hypertensive encephalopathy, strokes or neurogenic pulmonary edema resulted, thus leading to sudden death and heart failure [8,12,13].

Some patients with pheochromocytoma may be asymptomatic, and it is not until incidental findings in diagnostic images suggest the presence of such tumors. Precise identification of adrenal incidentalomas occurs in only 0.8–5% of cases; although diagnostic images can detect adrenal masses, differential diagnoses such as adrenocortical carcinoma can ensue. Thanks to widespread use of Computerized Tomography, reported incidence of adrenal incidentalomas and pheochromocytomas has improved [6,15].

Given their low incidence, indeed, many physicians have never seen a patient with this pathology, pheochromocytomas are difficult to diagnose, especially in cases of non-specific clinical symptomatology. Likewise, many are under-diagnosed, or diagnosed post-mortem. Therefore, it is important to suspect this pathology as a differential diagnosis in patients who conform to the abovementioned clinicalprofile [2,8].

In addition to the harm caused by pheochromocytoma secretion of different types of catecholamines, the tumor can also trigger a hyperglycemic crisis, resulting in altered insulin secretion as well as greater insulin resistance. This syndrome contributes to misdiagnosis with diabetes mellitus and diabetic ketoacidosis [14].

Warning signals that prompt biochemical study for this type of tumors include clinical suspicion based on unaccountable variations in blood pressure, excessive catecholamine levels and hypertensive crises under stimuli such as exercise or anesthesia, coupled with cardiovascular risks and complications that compromise patient’s life [1,2,9].

According to Guerrero et al., a direct relationship exists between tumor size and catecholamine production, a factor that bears no relation to clinical manifestations. Other studies have also described “clinically silent” pheochromocytoma, typified by elevated hormonal changes, unaccompanied by subsequent clinical signs. Furthermore, most giant pheochromocytomas will not produce the classic symptoms as described above [3,4,11].

The identification of catecholamine synthesis and secretion is one of the most important steps in diagnosing this type of tumor. However, catecholamine release may be minimal; whereas, constant metabolite release, for instance, metanephrine and normetanephrine, is common [1].

Plasma and urinary levels of catecholamine metabolites (metanephrine and normetanephrine) have shown high sensitivity and diagnostic specificity. It is important to take into account false positives, especially in patients undergoing dialysis or in those who have suffered sudden cardiovascular events. Likewise, the use of recreational drugs, such as cocaine or amphetamines, may produce similar symptoms [1,2,10].

Several studies have shown that amphetamine measurement in urine or plasma provides an excellent diagnostic tool, with plasma measurement demonstrating greater sensitivity and specificity [10].

Therefore, proper pheochromocytoma diagnosis depends upon correlating information from patient´s medical history from their clinical signs and symptoms and from their paraclinical and biochemical test results. It is important to keep in mind that in asymptomatic patients, even the lowest elevation of metanephrines in urine during a 24 -h period reflects catecholamine secretion suggestive of pheochromocytoma [16].

Testing for differentiation between catecholamine production due to autonomous nervous system activation and that due to tumor secretion most commonly employs the Clonidine Suppression Test. This test possesses high positive predictive value while allowing for the fact that a normal result does not exclude the presence of a pheochromocytoma. Depending on the pattern of catecholamine secretion, a pheochromocytoma can be sub-classified as noradrenergic, adrenergic or dopaminergic [8,10].

Precise tumor location, detailed biochemical studies and diagnostic imaging form the basis of successful pheochromocytoma diagnosis and treatment. Needless to say, accurately locating the tumor will determine any future surgical approach; and from a purely functional standpoint, it is requisite in evaluating possible metastasis [2].

Accurately locating a tumor relies upon Computerized Tomography (the study of diagnostic images produced by Magnetic Resonance Imaging), which is usually called for in specific cases of children, pregnant women or patients with suspected hereditary pathologies. Since there are various types of diagnostic images, in the case of the pathology under discussion MRI contributes not only to diagnosis but also to the evaluation of various scenarios for treatment and subsequent follow-up [8,17].

Surgery continues to be overwhelmingly preferred for pheochromocytoma treatment; despite the fact that the combined intra-and post-operative resultant mortality rate is a remarkably high 50%. Although complete resection of these tumors remains the only cure, potential cardiovascular risks such as hypertension crises or arrhythmias can occur when catecholamines are released during surgery [18,19].

Surgical pheochromocytoma resection can be a high-risk, life-threatening procedure that generating lethal hypertensive crises due to peripheral vasoconstriction and decreased intravascular volume. Complete preoperative management should anticipate complications that could arise from intravascular instability and fluctuating blood pressure, secondary to the handling of the intra-operative tumor. Therefore, it is essential that pheochromocytoma treatment be based upon a multidisciplinary medical approach that combines anesthesiology, internal medicine, cardiology and surgery [10,20].

Therefore, it is essential to stabilize patient´s blood pressure with an adrenergic alpha medication, such as prazosin, 1–2 weeks prior to surgery; and simultaneously, to be on the alert for any side effects, including orthostatic hypotension. Despite pharmacological management, due to release of catecholamines, hemodynamic instability is frequent during intra-operative tumor manipulation [10,21].

Since Gagner performed the first unilateral adrenalectomy with lateral decubitus, minimally invasive surgery for this pathology has become the gold standard. Nonetheless, only patients with large or difficult tumors should undergo laparotomy [10,23].

Given the anatomical location of the suprarenal glands, the upper retroperitoneal space, surgical approaches are broad, including transperitoneal, retroperitoneal and transthoracic approaches [24].

The literature describes four different laparoscopic adrenalectomy approaches: lateral or anterior transperitoneal; and lateral or posterior retroperitoneal. Lateral transperitoneal and retroperitoneal approaches are preferred because they are more direct and offer greater exposition [23].

However, choice of surgical approach depends on other factors, as well: tumor size and vascularization, signs of malignancy, presence of local invasion, and surgeon’s experience [6].

Minimally invasive surgery, as opposed to open surgery, entails many advantages, including less postoperative pain, less hospitalization, faster recovery and lower morbidity [25].

Laparoscopic adrenalectomy using either the retroperitoneal or the transperitoneal approach is the surgical method-of-choice for suprarenal tumors. Due to the limitations of the surgical area, surgeons prefer laparoscopic adrenalectomy to resect adrenal tumors of less than 7 cm diameter [22,27].

Despite breakthroughs in surgical methods and perioperative management, minimally invasive pheochromocytoma resection continues to be hazardous; mainly due to the contiguous risk of intraoperative hemodynamic instability. And, although many case reports may confirm the efficacy and safety of laparoscopic surgery; the debate goes on over whether or not it is appropriate for large tumors [26].

Indeed, most cases in the literature report on tumors that measure less than 10 cms where surgical choice, even for tumors under 6 cm, is also laparoscopic surgery. Furthermore, regardless of tumor size, laparoscopic resection is usually preferred when tumor mass is in close contact with the renal vein or inferior vena cava [27].

However, open surgery prevails if tumor malignancy is suspected, when tumor size surpasses 8 cms or where hypersecretion of multiple steroid hormones or clinical feminization characteristics are present [24].

In a retrospective study that evaluates the feasibility of laparoscopic adrenalectomy using the lateral retroperitoneal approach for pheochromocytoma greater than 6 cms, Chung HS, et al., showed this surgical technique to be a good option, regardless of tumor size, with adequate perioperative hemodynamic control, thus reconfirming preference for the laparoscopic approach in resecting large tumors [26].

Currently, use of the transperitoneal approach to treat lesions that measure more than 6 cms remains open to debate. This surgical method is widely accepted for treating small tumors, thanks to its resection and dissection capacity that offers a clear, although reduced, intraoperative visual field: whereas, open surgery still prevails as the first-line surgical approach for total resection of larger tumors [13,28,29].

In a retrospective cohort study comparing open adrenalectomy with transperitoneal laparoscopic adrenalectomy, Bai S, et al., evidenced lower incidence of both intraoperative hemodynamic instability and prolonged postop hypertension, greater recovery of postoperative ileus and lower cardiovascular morbidity among laparoscopic surgery patients whose tumors measured between 6 and 12 cms [29].

Likewise, Conzo G, et al., compared the transperitoneal approach to the retroperitoneal approach. They concluded that the former method offers more advantages, including direct access to the suprarenal gland and the suprarenal vein, which thereby eliminates the need to pass through the peritoneal cavity and move adjacent intra-abdominal organs [28].

However, to date, insufficient comparative studies exist that address the issue of which technique or standard method should be used to approach minimally invasive surgery for large tumors. Furthermore, profiling pheochromocytoma surgical treatment requires specific data because, as opposed to other tumors, a pheochromocytoma secretes excessive catecholamines, accompanied by high vascularization and possible adherence to adjacent organs, thus resulting in greater intra and post-operative complications, especially when tumor size surpasses 6 cms [29].

The gold standard for surgical approach in resection of large size pheochromocytomas has yet to be set; as numerous authors recommend a certain tumor size limit for laparoscopic approaches when treating pheochromocytomas.

Meanwhile, individual surgeons, who depend upon their unique backgrounds and individual learning curves, will continue to choose, on a case-by-case basis, the most effective and safest approaches for surgical treatment.

The advantages of minimally invasive adrenalectomy over open surgery include less postoperative pain; reduced hospitalization; decreased intraoperative hemodynamic instability, due to tumor manipulation, and lower cardiovascular morbidity, factors all of which mean that this method could become the most widely preferred for resecting large size pheochromocytomas.

Although pheochromocytoma is a rare pathology, specialists worldwide recognize it merits its own, specific diagnostic and therapeutic approaches; however, this tumor remains widely unknown in Colombia, where, furthermore, laparoscopic transperitoneal adrenalectomy for large size pheochromocytomas is extremely uncommon.

The purpose of this paper is to show how, despite the lack of resources and surgical experience in our country, it is possible to perform this type of minimally invasive surgery. We hope we have motivated surgeons, who face numerous obstacles, to recognize that professional and academic growth is best achieved by is sharing our knowledge that, in turn, will lead to better patient treatment in our local setting.

To date, no case description on this type of surgical procedure is available in Colombia. Similarly, in most developing countries, which face the same limitations as Colombia does, it is scarcely mentioned. Hopefully, sharing our clinical and surgical experience in dealing with laparoscopic transperitoneal adrenalectomy in large size tumors can contribute to future comparative studies. We are working towards consensus on a standard surgical approach for large pheochromocytomas that will take into account surgeorns´ learning curves, especially of those in developing countries, including Colombia, where medical centers often lack the requisite surgical tools and experience of to treat this rare pathology.

## Declaration of Competing Interest

The authors declare that they have no conflict of interest.

## Funding

No grants funded this study. Researchers donated office supplies and researcher time.

## Ethical approval

This case report was submitted for consideration, comment, guidance and approval to the Ethics Committee of the *Fundación Santa Fe de Bogotá Hospital* in Bogota, Colombia. Approved study will use medical history of established population. Said use will be confidential, and information contained therein shall be exclusively for research purposes. In compliance with the general provisions of Resolution No. 008430/ 1993 (October 3, 1993 of the Colombian Constitution - CC) whereby the scientific, technical and administrative standards for health research are established. The study of the diagnostic test is included within health research activities.

The privacy of the individual research subject will be protected (Article 8 - CC) by identifying him/her using codes. According to risk criteria set forth in Article 11 numeral A (CC), study is non- risk: research consists of collecting non-sensitive information from medical histories and aims to describe test behavior.

## Consent

In accordance with case report study guideline, patient’s verbal and written consent were requisite. Patient reviewed and clarified institutional consent of the *Fundación Santa Fe de Bogotá Hospital* which guaranteed patient’s medical history confidentiality his and anonymity. In patient’s written consent, dated September 21, 2018, approval was obtained for case report publication as well as for additional relevant images and information. Researchers will no share identifying and confidential patient data from case report.

## Author contribution

All Authors read and approved the manuscript.

**Roosevelt Fajardo (RF)**: Was the lead surgeon on transperitoneal laparoscopic adrenalectomy, interpreted the patient data and performed the conceptualization and supervision of the manuscript.

**Francisco Díaz C. (FD):** Was the second surgeon present during minimally invasive surgery, designed the methodology, analyzed the patient’s case and validated it.

**Nicole García C.** (**NG):** Was the principle manuscript author, presented the writing-original draft, carried out the process of visualization and research of data collection.

## Registration of research studies

Does not apply for this case report.

## Guarantor

Roosevelt Fajardo.

## Provenance and peer review

Not commissioned, externally peer-reviewed.
